# Training medical students as basic life support instructors: a demonstrative method pilot study

**DOI:** 10.3389/fmed.2025.1676697

**Published:** 2025-10-22

**Authors:** Pedro Fernández Florido, Francisco Manuel Parrilla Ruiz, Teresa Rodríguez Fernández de Simón, Lydia Álamo García, Gerardo Gómez Moreno, José Miguel Pérez Villares, Antonio Cárdenas Cruz

**Affiliations:** ^1^Department of Intensive Care Medicine, Virgen Macarena University Hospital, Seville, Spain; ^2^CriticalLab CTS 609 Research Group (PAIDI), TEC 23 IBS Granada Research Group, Granada, Spain; ^3^Emergency Department, University Hospital Clínico San Cecilio, Faculty of Medicine, University of Granada, Granada, Spain; ^4^Intensive Care Medicine Service, University Hospital Virgen de las Nieves, Granada, Spain; ^5^Department of Stomatology, Faculty of Dentistry, University of Granada, Granada, Spain; ^6^PAIDI CTS 654 Research Group, Graduate in Medicine, University of Granada, Granada, Spain; ^7^Head of the Intensive Care Medicine Department, University Hospital Virgen de las Nieves Granada, Granada, Spain; ^8^Intensive Care Medicine Service, University Hospital Virgen de las Nieves, Faculty of Medicine, University of Granada, Granada, Spain

**Keywords:** training, basic life support, cardiorespiratory arrest, students, demonstration method

## Abstract

**Introduction:**

Training the general population in basic life support is essential because it increases the likelihood that laypeople will perform high-quality cardiopulmonary resuscitation early on, which has a significant impact on patient outcomes. The university stage is a pivotal time to impart this knowledge to individuals outside the healthcare sector. This study aims to describe and analyses the extent to which students on the Medicine degree programme at the University of Granada have acquired competencies in teaching methodology applied to life support instruction. Additionally, the study aims to evaluate the potential academic and social impact of this training intervention.

**Methods:**

This is a prospective observational study targeting a selected group of medical students from the University of Granada. The students received specific training in teaching methodology applied to basic life support teaching and learning processes. The students' acquisition of various skills is assessed to determine their readiness to become trainers in this field.

**Results:**

A total of 89 students, all in their third to fifth year of university, received training in the methodology applied to teaching and learning processes in basic life support. This training used the different phases of the demonstrative method. Four training sessions were conducted, during which the phases of the demonstrative method were explained in detail and the students were given the opportunity to carry them out. The performance of the entire process was then assessed. As a result, over 89% of students successfully completed the phases of the demonstrative method.

**Discussion:**

Medical Degree students are capable of acquiring skills in teaching methodology to conduct Basic Life Support training courses for the general population. This is independent of the year in which they are in their training, as long as they have received theoretical training in this subject. Further studies are encouraged by these results, with the aim of extending this type of training to other medical education centers nationwide.

## 1 Introduction

Cardiac arrest (CA) is defined as the sudden, unexpected and potentially reversible cessation of the mechanical activity of the heart and spontaneous breathing. Due to its high morbidity and mortality rates, it has come to be considered a serious public health problem by the World Health Organization (WHO). In fact, in Spain alone, it causes up to 45,000 deaths annually. In Europe, the annual incidence of out-of-hospital CPA is reported to be between 67 and 170 per 100,000 inhabitants (1).

It is in out-of-hospital CRA that rapid intervention by those who witness the event is most important. The set of actions to be carried out by the people who witness the CRA until the arrival of healthcare personnel is known as Basic Life Support (BLS), which includes early recognition of the CRA situation and request for help, cardiopulmonary resuscitation (CPR) and, if necessary, defibrillation. All this, with advanced life support and post-resuscitation care by healthcare personnel, is known as the “Chain of Survival” (1). It is essential that these actions are carried out as quickly as possible because CRA is a time-dependent pathology; in other words, a delay in starting resuscitation maneuvers has a direct negative impact on the patient's prognosis. From all this, we can deduce the importance of the formal training of the general population in these techniques.

In Spain, the percentage of the population receiving BLS training is very small, unlike in other countries where learning these techniques is incorporated from primary and secondary education (2). Some studies estimate that up to 42% of the world's population may have received occasional BLS training at some point, but not in a formal way or with subsequent refresher courses, and there is enormous variability between geographical areas (3). Recent evidence also shows that the willingness to perform bystander CPR and the likelihood of providing high-quality resuscitation are strongly associated with systematic traning programmes introduced early in education systems (4). This has a direct impact on the prognosis of CRA (5). Indeed, the 2018 European Resuscitation Council guidelines emphasized the importance of community-wide approaches and innovative educational strategies to boost bystander CPR rates (6). In fact, there has been a call to modify the “Chain of Survival,” turning it into a “Cycle of Survival,” adding a specific link referring to the preparation of the general population in BLS techniques (7). The key points of BLS training for bystanders and non-health first responders are considered the following (8):

1. Increase the willingness to perform CPR.
2. Reinforce the chain of survival.
3. Teach resuscitation using feedback devices.
4. Spread resuscitation training over time (spaced education).
5. Maintain resuscitation competencies through frequent renofresher training.

There is much heterogeneity in terms of how to conduct this training, what content to teach and how to assess it. Despite this, instructor-led face-to-face courses were superior to other educational strategies employed (9). A recent study highlights that simulation-based training and structured evaluation tools enhance skill retention and teaching effectiveness when training laypeople in resuscitation (10). Furthermore, meta-analyses confirm that spaced education and practice with feedback significantly outperform traditional one-off training sessions in maintaining CPR quality over time (11).

The demonstrative method is one of the most widely used methods for developing practical workshops in recent years. This method allows students to practice the skills they need to acquire in a controlled environment designed specifically for this purpose, through simulation. The demonstrative method consists of different phases, which are:

1. Teacher presentation.
2. Presentation of the workshop, including its title.
3. Definition of the main objective of the workshop.
4. Performance of the technique to be taught in non-real time. At this point, the students can ask any questions they consider appropriate, interrupting the teacher's demonstration.
5. Performance of the technique in real time. At this point, no interruptions could be made. The aim is for the student to be aware of the effective time in which the technique can be performed.
6. Development of a summary of the key points of the workshop.
7. Development of feedback techniques. Allowing questions from learners again.
8. Invite learners to perform the technique.
9. Completion of a debriefing without judgement.

It should also be noted that, traditionally, BLS training has fallen on the healthcare providers as the ones in charge of transmitting it to the general population. However, we believe that two fundamental elements that may hinder this function should be taken into account: the shortage of healthcare personnel for such an ambitious objective, as well as the low level of training in teaching methodology to transmit these techniques in an appropriate manner. Furthermore, recent analyses suggest that the high workload of healthcare professionals and the limited teaching experience of many trainers could limit the scalability of community BLS training programmes (12).

Our study arose from the idea of trying to solve this problem by including medical students in the training of the general population in BLS techniques. These students are systematically trained in these techniques. In this way, thanks to the large number of students enrolled in the various medical schools, we can overcome the shortage of health care personnel who can extend BLS training to a larger percentage of the general population. Even so, there is still a problem: the lack of knowledge of the teaching methodology on the part of the students.

To fill this gap, we designed a training methodology course applied to the teaching of life support and aimed specifically at medical students who had previously received BLS training. In this way, it would be possible to enormously increase the number of the population reached by CPR training, since we would have a large number of medical school students with a profound knowledge of these techniques and who would also have acquired training in teaching methodology.

Therefore, the hypothesis of our study would be defined as follows: medical students, after receiving training in teaching methodology centered on the development of the demonstrative method, can participate as teachers in BLS courses in an adequate and tutored manner, being able to extend the training of the general population in these techniques. The demonstrative method is one of the most commonly used methods for the development of practical workshops.

## 2 Material and methods

### 2.1 Ethics committee approval

This study has a favorable report from the UGR research ethics committee with code 4036/CEIH/2024.

### 2.2. Study design

This is a prospective observational pilot evaluation of competency acquisition. It is based on the design of a training course in teaching methodology, with the subsequent aim of analyzing the acquisition of competencies in BLS training methodology by medical degree students.

The study was conducted at the Faculty of Medicine of the University of Granada (UGR). As part of their undergraduate medical curriculum, all students had previously completed formal Basic Life Support (BLS) training. Subsequently, a specific course on teaching methodology applied to life support education was designed and implemented following a selection process based on voluntary expression of interest. A total of 89 students participated in this training intervention. All students who wished to participate were able to do so on a voluntary basis. It is important to note that participation in these workshops was entirely voluntary, and no academic bonus or other rewards were offered.

### 2.3 Course design

The training was given to the students of the Faculty of Medicine of the UGR by health personnel specialized in the care of critical patients in general and in the care of CPR in particular. It should be noted that, although these instructors received training on the different aspects of the demonstration method, they have no prior specific pedagogical training.

The courses were organized into groups of 25 students. Two instructors worked with each group. The course lasted approximately 3 h.

The course was divided into two didactic units:

– Didactic unit A: Basic CPR workshop.
– Didactic unit B: Semi-automatic external defibrillation (DESA) workshop.

The methodological resource used was the demonstrative method. Students did not receive any materials prior to the course. It should be noted that the course primarily assesses the acquisition of teaching skills for conveying knowledge in BLS. It is therefore a course primarily focused on pedagogical training and teaching methodology.

First, in order to carry out this training, the teachers carried out the different phases of the demonstrative method, with the aim of ensuring that the students assimilated the different phases to be carried out in order to adequately transmit their knowledge of CPR and BLS.

– During didactic unit A, basic CPR was performed sequentially, based on the demonstrative method:

○ Check the level of consciousness by shouting and shaking.
○ Call for help.
○ Clear the airway using the forehead-chin maneuver and check if the patient is breathing using the see, hear and feel maneuver.
○ Activate the emergency service and request an AED.
○ Properly locate the point of cardiac massage and perform the proper cardiac massage technique.

– In unit B, which focuses on the handling of the AED, the handling of the device was practiced:

○ Requesting an AED.
○ Switching on the device.
○ Place the patches at the point indicated on the device itself.
○ Follow the instructions provided.
○ Interspersing continuous, quality chest compression with the AED commands.

After this demonstration by the teachers, the students carried out all the phases of the demonstration method one by one, just as they would do in a course at training in CPR and BLS for the general population. In this way, all the students assumed the role of teacher or instructor during the course. It is during this part of the training workshop that the course instructor also had the opportunity to assess whether the students included in the project had the knowledge of BLS techniques well internalized and structured, with the possibility of transmitting them later on. The trainees more than fulfilled this requirement, as they had all received formal training during their university education.

Given that a total of 89 students participated, and each course consisted of 25 students, it was necessary to run four courses, which occurred between February and April 2025. They took place in the facilities of the Faculty of Medicine of the UGR, being able to use the advanced clinical simulation laboratories available at the same.

The materials used were basic CPR training simulators and practice AEDs.

### 2.4 Variables to be analyzed

The variables to be analyzed in this study were subdivided into two categories: sociodemographic variables and academic variables.

With regard to the sociodemographic variables, the following were included:

○ Age.
○ Year of academic training.
○ Sex
○ Whether they had ever been involved in emergency or CRA care.

With regard to the academic variables, the following were analyzed:

○ The aptitude after performing the different phases of the demonstration method.
○ The students' capacity for self-criticism.

To analyse the suitability of the different phases of the demonstration method, we designed a data collection table on the evaluation sheet detailing these phases. The data collection sheet is also included as Supplementary material. Since two teachers participated in each course, both teachers evaluated each student. If there were discrepancies in the evaluation of an item, the lower of the two grades was selected.

Note that all student evaluations were anonymous, using a pseudo-anonymization process, so that there was no influence on the evaluation or interpretation of the data.

Two aspects of each of these phases were evaluated: whether it adequately met the objective of the item and the form of expression (both verbal and non-verbal) in each of them. Within the form of expression, the following elements were assessed:

○ Verbal language control:

■ Verbal fluency
■ Mental fluency
■ Mastery of emphasis or if it was monotonous.
■ Organization and transmission of ideas

○ Control of the vocal language:

■ Adequate volume
■ Good diction
■ Adequate speed
■ Adequate pitch
■ Correct timbre
■ Use of silences

Control of the body language:

■ Facial expression
■ Body expression
■ Control of the stage (movement)
■ Use of distances
■ Use of the gaze

○ Control of situational language, mainly focused on stage control.

Thus, the performance of each phase of the demonstrative method was categorized as follows:

○ Performed the item correctly: the learner met the objective of the phase to be performed, with a good capacity for expression.
○ The candidate performed the task correctly, but with minor errors: the objective was achieved, albeit with inadequate verbal or non-verbal expression. Therefore, we consider errors in expression to be minor errors, provided that the task objectives are met.
○ The candidate made major errors and had to repeat the entire sequence. A major error is defined as failing to meet the task objective.

The students' ability to be self-critical and to detect any mistakes they may have made during the exercise was also assessed, as this would make it easier for them not to make them again in the future, as they themselves were the detectors of these mistakes. The instructors guided this process, encouraging the students to express how they felt and to identify their strengths and weaknesses. The students completed a self-report guided by the instructors.

### 2.5 Data analysis

The statistical study was subdivided into two different phases. First, a descriptive analysis was carried out of all the variables under study, both of the affiliation data and demographic characteristics of the population included in the study, as well as of the skills to be assessed during the training process of the trainees. Second, an inferential analysis was carried out, comparing the different study variables with each other. For the comparison between the dichotomous qualitative variables, a contingency table of the variables to be compared was obtained, subsequently applying the Chi-Square test. For the comparison of a dichotomous qualitative variable with another polytomous qualitative variable, or for the comparison of two polytomous qualitative variables with each other, the contingency tables were obtained first, then the Chi-Square test was applied, as well as the Bonferroni correction and the corrected standard-residuals. Statistical significance was defined as *p* < 0.05 for all comparative studies of variables.

For the statistical analysis, the statistical programme IBM^®^ SPSS 28.0 for MAC^®^ was used after dumping the information obtained in the database completed during the study.

## 3 Results

Eighty-nine (86.4%) of the students who volunteered to participate completed the training course in BLS teaching methodology. Fourteen students (13.6%) withdrew due to scheduling conflicts.

– Table 1 shows the demographic characteristics. Participants' ages ranged from 20 to 46 years, with an average age of 22 years. Most were female (63; 70.8%), while 26 were male (29.2%). By year of study, the largest group was in the fourth year (45 students; 50.6%), followed by the third year (40 students; 44.9%), with a smaller proportion in the fifth year (four students; 4.5%). First- and second-year students were excluded because they had not yet received training in the relevant techniques. Sixth-year students were excluded due to their imminent graduation.
– Previous exposure to emergencies (Table 2): Analysis of the association between academic year and previous experience of an emergency or cardiopulmonary arrest revealed statistically significant differences. Pearson's chi-squared test yielded a significant result [χ^2^(2) = 7.292; *p* = 0.026], indicating that the distribution of responses was not independent of the academic year. Similarly, the likelihood ratio yielded a significant value [χ^2^(2) = 8.162; *p* = 0.017]. Examining the corrected residuals revealed the direction of these differences: in the third year, there were more students without experience and fewer students with experience than expected, while in the fourth year, the opposite was true. In the fifth year, the observed and expected values were very similar, with no significant differences. Regarding the magnitude of the association, the symmetric measures indicated Cramer's Phi and V values of 0.286 (*p* = 0.026), which are interpreted as indicating a low-to-moderate-intensity significant relationship. In summary, the results show that the academic year influences the probability of having had experience of an emergency or cardiopulmonary arrest. Specifically, fourth-year students report previous experiences of this type more frequently, while the lowest proportion is observed in the third year. No notable differences from the expected values were found in the fifth year.
– Performance in the phases of the demonstrative method.

○ Teacher presentation: Almost all students (98.9%) performed adequately; only 1.1% performed poorly.
○ Workshop presentation: 81 students (91%) performed correctly, while five (5.6%) made minor errors and three (3.4%) omitted a step, requiring the sequence to be repeated.
○ Definition of objectives: This was correctly done by 84.3% of students; 7.9% made minor errors, 2 (2.2%) made major errors and 5 (5.6%) omitted it.
○ Technique in non-real time: Performed correctly by 78 students (87.6%); 10 students (11.2%) made minor errors, and one student (1.1%) did not perform it.
○ Encouraging questions during non-real time: 83 students (93.3%) encouraged questions; 5 (5.6%) made minor errors and 1 failed to allow questions.
○ Technique in real time: Correctly executed by 62 students (69.7%); 23 students (25.8%) made minor errors and four students (4.5%) did not perform it.
○ Summary of key points: It was performed correctly by 55 students (61.8%), 25 students (28.1%) made minor errors and 9 students (10.1%) omitted it.
○ Final feedback: Conducted appropriately by 72 students (80.9%); nine (10.1%) made minor errors and eight (9%) omitted it.
○ Motivation for practice: Correctly completed by 76 students (85.4%); eight (9%) made major errors and five (5.6%) omitted it.
○ Self-criticism: 75 students (84.3%) correctly identified their mistakes, six (6.7%) only partially identified them and eight (9%) were not self-critical.

– Inferential analysis: Two comparisons were performed:

○ By sex: No statistically significant differences were found between males and females in any of the phases of the demonstrative method; performance was similar in both groups.
○ By year of study (Table 3): Differences were only observed in the section on summarizing and defining key points. Analysis of the relationship between academic year and the ability to identify key points in the CPR course revealed statistically significant differences. Both Pearson's chi-square test [χ^2^(4) = 16.642; *p* = 0.002] and the likelihood ratio test [χ^2^(4) = 19.749; *p* < 0.001] yielded significant values, confirming that the distribution of responses differs according to academic year. Examination of the corrected residuals revealed significant differences. In the third year, there were more cases than expected in the “Some failure” category (residual = +1.8), while “Does not perform” was below expectations and there was a slight over-representation in “Perfect.” In the fourth year, the opposite occurred: fewer cases than expected in “Some failure” (−2.7) and more in “Does not perform” (+3.1), although “Perfect” was in line with expectations. In the fifth year, there was an overrepresentation in “Some failure” (+2.1) and fewer than expected in “Perfect” (−1.5). Regarding the magnitude of the association, the symmetric measures showed Phi values of 0.432 and Cramer's V values of 0.306 (*p* = 0.002), indicating a significant moderate-intensity association. In summary, the results show that the academic year affects students' ability to identify key points in the CPR course. Third-year students performed best, with a high proportion of responses in the “Perfect” category and a low frequency of “Does not perform.” In contrast, in the fourth year, a higher proportion of students do not adequately define key points, while in the fifth year, the proportion of students with some errors increases and the proportion of perfect performances decreases.

**Table 1:** Participant demographics.

**Demographic Variable**		**Frequency**	**Percentage**
Age	20	30	33.7
	21	26	29.2
	22	20	22.5
	23	4	4.5
	25	1	1.1
	27	2	2.2
	29	3	3.4
	32	1	1.1
	36	1	1.1
	46	1	1.1
Course	3○	40	44.9
	4○	45	50.6
	5○	4	4.5
Gender	Female	63	70.8
	Male	26	29.2
Have you ever been involved in emergency or PCR care?	No	74	83.1
	Yes	15	16.9

It provides a detailed profile of the participating students, differentiating them by age, year group, gender, and previous experience of responding to an emergency or cardiac arrest. The frequency and percentage of each characteristic relative to the total number of students is indicated.

**Table 2:** Comparative table of previous participation in emergency or CPR care by course.

**Year**		**Have you ever been involved in emergency or CRA care?**		
		**No**	**Yes**	**Total**
3	Count	38	2	40
	of total	42.7%	2.2%	44.9%
4	Count	33	12	45
	% of total	37.1%	13.5%	50.6%
5	Count	3	1	4
	of total	3.4%	1.1%	4.5%
Total	Count	74	15	89
	of total	83.1%	16.9%	100%

Fourth-year students had participated in CPR or emergency care the most previously (50.6%), compared to third- and fifth-year students (44.9% and 4.5%, respectively).

**Table 3:** Comparative table showing summary completion and definition of key points, categorized by course.

**Year**		**Summary with definition of key points**			
		**Some failure**	**Not done**	**Perfect**	**Total**
3	Count	15	0	25	40
	% of total	16.9%	0.0%	28.1%	44.9%
4	Count	7	9	29	45
	% of total	7.9%	10.1%	32.6%	50.6%
5	Count	3	0	1	4
	of total	3.4%	0.0%	1.1%	4.5%
Total	Count	25	9	55	89
	of total	28.1%	10.1%	61.8%	100%

Fourth-year students performed best on this demonstrative method item (50.6% in total), compared to students on other courses. Additionally, this course has the highest proportion of students who completed it without errors (32.6% of the total) (*p* = 0.02).

Table 4 presents the results of the statistical analysis comparing the different phases of the demonstrative method among students of different genders and academic years.

**Table 4:** This table compares the different phases of the demonstrative method with gender, and shows the progress of different students.

**Comparison**	***p-value***	**Phi**	**V de Cramer**
Gender ^*^ Teacher presentation	0.117	−0.166	0.166
Gender ^*^ Presentation of the workshop, including its title	0.888	0.052	0.052
Gender ^*^ Definition of the main objective of the workshop	0.263	0.212	0.212
Gender ^*^ Performance of the technique to be taught in non-real time	0.242	0.179	0.179
Gender ^*^ Students can ask any questions	0.722	0.086	0.086
Gender ^*^ Performance of the technique in real time	0.242	0.179	0.179
Gender ^*^ Development of a summary of the key points of the workshop	0.369	0.150	0.150
Gender ^*^ Development of feedback techniques	0.931	0.040	0.040
Gender ^*^ Invite learners to perform the technique	**0.040**	0.269	0.269
Gender ^*^ Self-criticism	0.547	0.116	0.116
Course ^*^ Whether they had ever been involved in emergency	**0.026**	0.286	0.286
Course ^*^ Teacher presentation	0.538	0.118	0.118
Course ^*^ Presentation of the workshop, including its title	0.148	0.276	0.195
Course ^*^ Definition of the main objective of the workshop	**0.041**	0.384	0.272
Course ^*^ Performance of the technique to be taught in non-real time	0.772	0.142	0.101
Course ^*^ Students can ask any questions	0.118	0.288	0.203
Course ^*^ Performance of the technique in real time	0.086	0.303	0.214
Course ^*^ Development of a summary of the key points of the workshop	**0.002**	0.432	0.306
Course ^*^ Development of feedback techniques	0.466	0.201	0.142
Course ^*^ Invite learners to perform the technique	0.051	0.325	0.230
Course ^*^ Self-criticism	0.345	0.224	0.159

Those with a *p*-value of less than 0.05 are highlighted.

## 4 Discussion

According to the World Health Organization (WHO), CRA is considered a serious public health problem, mainly due to its high morbidity and mortality, as well as the fact that it is a time-dependent pathology (15). The time to initiate BLS and CPR is one of the main elements of improvement in the care of this patient profile. This is especially relevant in out-of-hospital CPR, where the first care should be provided by bystanders, whether they are healthcare or non-healthcare providers (16). To increase the likelihood of good initial care by these bystanders, while the Out-of-Hospital Emergency Services are on the scene, it is essential that the public is aware of BLS techniques. For proper care of a patient in CPR by a bystander, the bystander must be able to identify the CPR situation, call for help, initiate CPR maneuvers and use an AED (17).

The importance of training the general population in CPR and BLS for the first care of the CRA patient has been described. In fact, bystander CPR can double or even triple the survival rate of CPR (18). Thanks to training, it has been shown that the non-healthcare populations who witness such an event attend to the patient on a greater number of occasions and do so with greater diligence and safety. They are also better able to recognize the CRA situation and act more quickly (19). This is not only true among the general population but also among the healthcare workers themselves after training in these techniques (20). Moreover, this training has direct implications not only at the attitudinal level but also at the emotional level. In a study carried out by Mausz et al. (21), it is clear that people who have attended a CPR suffer episodes of anxiety, anguish, insomnia, etc., symptoms that can be similar to those suffered by a person with post-traumatic stress syndrome. After undergoing CPR training, the intensity and frequency of these symptoms decreased notably, and they even stated that they felt prepared to act again if they witnessed another event with similar characteristics.

The consensus on BLS training in the general population is contained in the recommendations established by the European Resuscitation Council (ERC) and the American Heart Association (AHA). Both institutions stress the importance of implementing formal BLS training to ensure the training of school-age children, with the aim of developing a predisposition and aptitude for CPR (22, 23).

However, the percentage of people worldwide who have received training in this area is not high enough, and there are discordant data between different geographical areas (3). This means that only 35%−45% of patients who suffer an out-of-hospital CRA receive initial care from people who witness the event (1, 3). Specifically in Spain, this is an area where we still have enormous room for improvement, with approximately 30,000 out-of-hospital CRAs occurring in our country each year (24).

With the intention of acting on this problem at the population level and with the aim of having a direct impact on the prognosis of the patient who suffers a CRA, healthcare personnel have always been heavily involved in the training of the general population in BLS. However, healthcare professionals alone cannot reach the whole of society, and this is one of the main limitations when trying to implement a formal BLS training plan for the general population (25). The implementation of training in teaching methodology, together with the knowledge that medical students receive during their university training, allows, as demonstrated in our work, students to be fully capable of participating as teachers in formal BLS training courses for the general population, always under the supervision of a healthcare professional with certified teaching experience. Previous experiences support our hypothesis and have already proven the ability of medical students to transmit BLS knowledge to other healthcare professionals and to the general population. Perkins et al. (26) developed a BLS training programme delivered by medical school students to students with other degrees, such as physiotherapy, dentistry, and nursing. These students who delivered the workshops were previously trained as BLS instructors. Following the workshops given by the medical students, the students who had attended the workshop were evaluated, with a success rate of 99%. The workshop was very well received by the students who received it, who were highly satisfied with the training. In addition, most trainees reported that they would be able to act on a PCR after receiving the workshop. Toner et al. (27) also conducted a study in which primary school teachers were trained by medical students. Subsequently, these teachers passed on the acquired knowledge to pupils aged 10–12 years, who were evaluated. Most of the students passed the evaluation, demonstrating that medical students can transmit BLS knowledge so effectively that even the general, non-healthcare population can in turn pass on the knowledge they have been taught.

When organizing a training course in teaching methodology for undergraduate medical students, it should be borne in mind that they may have received BLS training in different ways. Both the studies mentioned above (26, 27) do not clarify the teaching methodology used. It is true that there is no clear consensus on the best way to transmit BLS knowledge, and there is no evidence in the literature that any one method has been shown to be superior to the others (28). In our study, the demonstrative method was used as the methodological basis for the development of the clinical simulation, with students being able to practice the skills to be acquired in a controlled environment specifically designed for this purpose. This active method is superior to other more traditional teaching methodologies, especially regarding the transmission of procedural skills. Thus, the study by Evans et al. (29) compares the acquisition of competences in a skills workshop with the acquisition in a theoretical class, demonstrating greater knowledge in students who learn by doing. Aspegren's study (30) reached a similar conclusion, finding that more traditional and theoretical teaching methods, such as the expository method, are less effective than teaching through demonstrative methods using simulations. Therefore, there is clear evidence that medical educators should continue to use demonstrative, simulation-based and hands-on methods. Skills workshops should be increased, to the detriment of lectures and master classes, where passive information acquisition takes place and where the learner is not the focus of the training activity (31).

The teaching methodology based on simulation, as well as being more effective as previously mentioned, also has the advantage that the assumptions generated for the demonstration are controllable, flexible and can be adapted to the needs of the students, and can also be repeated if the student needs more practice to assimilate all the necessary knowledge and skills (32). However, they also have their disadvantages, in that many learners may find simulation learning challenging (33). This reduces their motivation to attend these workshops, as it is more convenient for the student to attend a master class, in which they play a much more passive and less participatory role. This should be taken into account by educators, who should adapt courses and workshops to create an atmosphere of trust, making them more individualized training, which can increase the participation and predisposition of the student (34).

Specifically in BLS training, the demonstrative method has also been shown to significantly increase the ability to apply the techniques learned (35). In a study by Bylow et al. (36) compared instructor-led BLS courses, using hands-on techniques, with self-study courses, following the provision of study materials. Instructor-led courses, using demonstration and simulation, demonstrated greater competence acquisition in the immediate post-course evaluation than the more theoretical courses. However, in the 6-month evaluation, the two teaching methodologies showed a similar rate of forgetting. This again highlights the importance of refresher sessions, which result in longer retention of practical BLS knowledge and skills.

Undergraduate medical students receive scheduled BLS instruction during their training as future physicians. These structured and scheduled courses significantly improve the acquisition of BLS skills (37). Even at the beginning of their university career, students show a greater knowledge of these techniques than the general population (38). However, this knowledge naturally increases and improves significantly after training. However, practical skills and theoretical knowledge are acquired more effectively if the trainees also try to pass on these skills afterwards. Geraldo Veloso et al. showed in their study that students who were required to pass on this knowledge after learning BLS showed a higher acquisition of BLS skills. This shows that passing on knowledge, in this case BLS, can be an effective learning methodology, as well as helping to disseminate these important skills to the general population (39). Breckwoldt et al. (40) compared three different methodologies of BLS training for fifth year medical students: traditional theoretical courses, theoretical training and subsequent practice with an out-of-hospital emergency department, and a third group that would learn BLS and then pass it on to students in a school. After the training, the students in the three groups were assessed for their knowledge of these techniques. There was statistically significant evidence in favor of the third group, where fewer relevant errors were made, as well as a higher score on the practical assessment. There were no significant differences in the theoretical assessment between the three groups. This agrees with our experience, as we have observed that, when performing the demonstrative method methodology in the workshop, the students had a deep and advanced knowledge of BLS.

It should also be noted that the benefits of training students in teaching methodology are widely known. In general, when a student is trained in communication and knowledge transfer skills, he/she begins to feel more confident when communicating with students or other professionals and has better organizational and knowledge management skills (41).

Furthermore, it is worth highlighting the ability of the undergraduate medical students participating in the study to perform self-criticism. This is fundamental, as it is directly related to their involvement in this teaching methodology, as well as to their interest in training and in better transmitting their knowledge of BLS. If students are able to recognize their mistakes, it means that they have understood the essence of the demonstrative method, that they are involved in this task of expanding their health knowledge and that, once they have detected possible mistakes, it will be more difficult for them to make them again.

We believe that the results obtained in our work are fundamental to change the paradigm of BLS training for the general population. We have always tried to ensure that trained healthcare personnel make knowledge of these techniques available to the general population, either in the hospitals themselves or in offices, educational centers, etc. However, it must be borne in mind that the number of healthcare workers is limited and that the work and care load they have to cope with is also high, making it even more difficult to carry out these necessary training activities. However, adding the efforts of medical students to the objective of spreading this knowledge would mean a great increase in the number of staff who could carry out these activities, thus multiplying the number of courses and workshops to be held.

Moreover, since each of these students has a different family and social environment, the transmission of this knowledge can be further extended, as more training courses could be organized in the workplaces or study places of their families and friends. It is also important to note that when training younger people, they will always feel more comfortable and closer, in a more trusting environment, with other trainees of a similar age to themselves, which can also make a difference in learning compared to older and perhaps more distant health workers, with whom this relationship of mutual trust is not established. This becomes even more important when it is pointed out that learning these techniques is more common in young, university-educated people (13, 14). It would therefore be particularly interesting to carry out this training in a regulated manner at the university level.

It should be noted that this work is fully reproducible. The demonstrative method is a method for transmitting knowledge that is mainly used in workshops or practical training, the usefulness of which is widely described. The data collection sheet is also easy to design, with the aim of assessing whether the trainees who will later become BLS instructors are capable of transmitting this knowledge.

Despite all of the above, our study is not without limitations. First, we believe that the fact that the trainees voluntarily participated may be biased because they are the ones most probably interested in training and teaching and, therefore, to have better results. However, we consider that teaching and training in BLS and CPR to the general population is a field that requires great commitment on the part of the person involved. Therefore, this interest and predisposition for teaching is necessary in order to be able to transmit this knowledge to the general population. To limit selection bias, it would be interesting to deliver this training in a regulated manner within the selected Medicine Degree courses, assessing participants' ability to acquire BLS training skills regardless of predisposition. However, to ensure the knowledge is passed on effectively, it would be best for the training to be delivered by staff directly involved in the subject, as this can influence the quality of subsequent training. It is important to emphasize that the students included in our study are students who have already received formal BLS training during their medical degree. Another limitation of our study is that it was conducted solely within a medical school. It would therefore be interesting to extend the study to other faculties, as this would make it easier to replicate in other settings. This would also enable us to investigate whether differences in BLS training between medical schools influence the acquisition of methodological skills.

We also believe that it would be interesting to expand this work by evaluating the retention of these methodological skills several months after the training is received, and by organizing training courses led by the previously trained students. This would enable us to assess the BLS knowledge acquired by the students' own pupils. To this end, an assessment could be carried out on the population that has received the courses, both during the workshop and several months later, once students have completed them. In this way, it would also be possible to analyse the forgetfulness curve of the population that receives training from the medical students. Furthermore, it could be compared with the results determined similarly also in the general population that had received workshops given by health personnel. This is particularly important as it is known that people who receive training but do not undergo frequent training suffer a significant deterioration in their BLS skills after 3–6 months (28). This is why workshops to recall and review all the information are considered essential (42).

Despite our study, there is still much work to be done. Although the collaboration of medical students would be an invaluable aid in the training of the general population in BLS, it is still insufficient to approach the already utopian goal of ensuring that 100% of the population knows how to act in the event of CRA. To this end, we believe that another line of research, on which we are already working, would be the training of non-health professionals who, after instructing them in BLS techniques, can transmit this knowledge to a wider population. In this sense, we believe that a key element of society that can reach a significant part of the population is the university teaching sector, on which we have decided to focus our future interventions.

## 5 Conclusion

### 5.1 Main conclusion

Students of the Degree in Medicine at the University of Granada who receive training in teaching methodology using the demonstrative method and clinical simulation are qualified to act as teachers in training actions related to BLS for the general population.

Further studies are needed in this area, particularly those involving training these students to work with the general non-healthcare population, to confirm these results in practice.

### 5.2 Secondary conclusions

Increasing society's knowledge of BLS and CPR is essential to improve the prognosis of CPR, both in terms of mortality and morbidity. Therefore, it should be a priority objective of our healthcare system.

The incorporation of medical students in the training of the general population in BLS techniques could have a great impact on increasing the percentage of the population with access to these techniques. It should not be forgotten that rapid action by bystanders to a CRA can be the difference between the survival and non-survival of the patient. Furthermore, not only this, but even if the patient survives, quality CPR and adequate BLS can mean that the patient has no or minimal sequelae. Conversely, if these techniques are not performed properly, even if the patient recovers spontaneous circulation, he or she may have totally disabling sequelae.

The ability of medical students to transmit BLS knowledge after receiving training in teaching methodology is independent of gender, as well as the university course they are in, as long as they have already received formal training in this subject.

Our aim is that this work will help other professionals and other medical schools in other universities to train their students in teaching methodology, so that they can also be included as qualified personnel to conduct BLS training sessions. Likewise, once this has been done, courses and workshops given by these students should be promoted to the non-healthy population.

## Data Availability

The raw data supporting the conclusions of this article will be made available by the authors, without undue reservation.
