# Supplementary material for: Training medical students as basic life support instructors: a demonstrative method pilot study
Source: Front Med (Lausanne). 2025 Oct 22;12:1676697. doi: 10.3389/fmed.2025.1676697 (PMC12586963; doi:10.3389/fmed.2025.1676697)
Supplement: Supplementary file 1 [file Data_Sheet_1.pdf]

Supplementary Material

Figure S1. Data collection sheet for student assessment. The different stages of the demonstrative method that must be completed are specified, along with an indication of whether or not they have been completed, and if so, whether they have been completed perfectly (column A, as indicated in the legend), whether there are some minor errors (column B), or whether there are serious errors and the stage must be repeated (column C).

EVALUATION SYSTEM FOR THE SVB TRAINERS COURSE [redacted]

ASSESSMENT OF TEACHING SKILLS. LIFE SUPPORT TECHNIQUES WORKSHOP (ASSESSMENT OF THE DEMONSTRATION METHOD)

DEMONSTRATION METHOD	YES	NO	SCORE		
			A	B	C
1. ADEQUATE PRESENTATION OF THE TEACHER					
2. PRESENTATION OF THE WORKSHOP: TITLE					
3. DEFINITION OF OBJECTIVES (adaptation of the objectives to the type of students)					
4. CARRYING OUT THE TECHNIQUE IN NON-REAL TIME					
5. ALLOWS THE STUDENT TO CONSULT ALL THE DOUBTS					
6. CARRYING OUT THE TECHNIQUE IN REAL TIME					
7. SUMMARY WITH DEFINITION OF KEY POINTS					
8. IMPLEMENTATION OF FEEDBACK TECHNIQUES					
9. INVITATION TO STUDENTS TO PERFORM THE TECHNIQUE					
10. SELF-CRITICS					

- NAME AND SURNAME STUDENT: (NAME SURNAME SURNAME SEX AGE YEAR OF THE GRADE HE/SHE IS STUDYING)
- SEX:
- AGE:
- COURSE:
- HAVE YOU EVER PARTICIPATED IN EMERGENCY OR PCR CARE?

A: YOU DO IT PERFECTLY
B: HAS SOME FAILURES BUT NOT SERIOUS
C: HAS SERIOUS FAULTS AND MUST REPEAT IT

Table S1. The percentage of female and male participants is presented relative to the total number of participants. The valid percentage is also presented, with no deductions, as there were no losses in our study.

		Frequency	Percentage	Valid percentage	Cumulative percentage
Valid	Female	63	70,8	70,8	70,8
	Male	26	29,2	29,2	100,0
	Total	89	100,0	100,0	

Table S2. We describe the percentage of students who had previously attended an emergency or CPR training session. We also describe the valid percentage without losses as there were none in our study.

		Frequency	Percentage	Valid percentage	Cumulative percentage
Valid	No	74	83,1	83,1	83,1
	Yes	15	16,9	16,9	100,0
	Total	89	100,0	100,0	

Table S3. We describe the percentage of students who give an adequate presentation of the teacher, differentiating between those who make some errors and those who make none. We also describe the valid percentage without losses, as there were none in our study.

		Frequency	Percentage	Valid percentage	Cumulative percentage
Valid	Minor errors	1	1,1	1,1	1,1
	Correctly	88	98,9	98,9	100,0
	Total	89	100,0	100,0	

Table S4. The table shows the percentage of students who presented the workshop perfectly (91%), those who made mistakes (5.6%), and those who did not do so (3.4%). The valid percentage is also described without losses as there were none in our work.

		Frequency	Percentage	Valid percentage	Cumulative percentage
Valid	Minor errors	5	5,6	5,6	5,6
	Omitted	3	3,4	3,4	9,0
	Correctly	81	91,0	91,0	100,0
	Total	89	100,0	100,0	

Table S5. Only 5.6% of students do not define the objectives. 84.3% do so correctly.

		Frequency	Percentage	Valid percentage	Cumulative percentage
Valid	Minor errors	7	7,9	7,9	7,9
	Major errors	2	2,2	2,2	10,1
	Omitted	5	5,6	5,6	15,7
	Correctly	75	84,3	84,3	100,0
	Total	89	100,0	100,0	

Table S6. Eighty-seven point six per cent of students performed the technique perfectly in non-real time, compared to 11.2 per cent who made some mistakes and 1.1 per cent who did not perform it.

		Frequency	Percentage	Valid percentage	Cumulative percentage
Valid	Minor errors	10	11,2	11,2	11,2
	Omitted	1	1,1	1,1	12,4
	Correctly	78	87,6	87,6	100,0
	Total	89	100,0	100,0	

Table S7. Of the total number of participating students, only 1.1% did not allow questions. 5.6% made some errors, while 93.3% performed it perfectly.

		Frequency	Percentage	Valid percentage	Cumulative percentage
Valid	Minor errors	5	5,6	5,6	5,6
	Omitted	1	1,1	1,1	6,7
	Correctly	83	93,3	93,3	100,0
	Total	89	100,0	100,0	

Table S8. The percentage of students who performed the technique perfectly in real time (69.7%) is shown. 4.5% did not perform the technique, while 25.8% made some errors.

		Frequency	Percentage	Valid percentage	Cumulative percentage
Valid	Minor errors	23	25,8	25,8	25,8
	Omitted	4	4,5	4,5	30,3
	Correctly	62	69,7	69,7	100,0
	Total	89	100,0	100,0	

Table S9. The summary with key point definitions was not performed by 10.1% of students. Meanwhile, 28.1% performed it with some errors, and 61.8% performed it perfectly.

		Frequency	Percentage	Valid percentage	Cumulative percentage
Valid	Minor errors	25	28,1	28,1	28,1
	Omitted	9	10,1	10,1	38,2
	Correctly	55	61,8	61,8	100,0
	Total	89	100,0	100,0	

Table S10. This table describes the percentage of students who performed the feedback techniques without errors (80.9%), with minor errors (10.1%), or did not perform them (9%).

		Frequency	Percentage	Valid percentage	Cumulative percentage
Valid	Minor errors	9	10,1	10,1	10,1
	Omitted	8	9,0	9,0	19,1
	Correctly	72	80,9	80,9	100,0
	Total	89	100,0	100,0	

Table S11. Of the 89 students in total, 76 (85.4%) invited their classmates to perform the technique without error, 5 (5.6%) did not perform it, and 8 (9%) performed it with some errors.

		Frequency	Percentage	Valid percentage	Cumulative percentage
Valid	Minor errors	8	9,0	9,0	9,0
	Omitted	5	5,6	5,6	14,6
	Correctly	76	85,4	85,4	100,0
	Total	89	100,0	100,0	

Table S12. Eighty-four per cent of students performed self-criticism adequately, compared to 9 per cent who did not.

		Frequency	Percentage	Valid percentage	Cumulative percentage
Valid	Minor errors	6	6,7	6,7	6,7
	Omitted	8	9,0	9,0	15,7
	Correctly	75	84,3	84,3	100,0
	Total	89	100,0	100,0	

Table S13. This table describes the percentage of students belonging to each year group. 44.9% belong to Year 3, 50.6% to Year 4 and 4.5% to Year 5.

		Frequency	Percentage	Valid percentage	Cumulative percentage
Valid	3	40	44,9	44,9	44,9
	4	45	50,6	50,6	95,5
	5	4	4,5	4,5	100,0
	Total	89	100,0	100,0	

Table S14. describes the mean, median, standard deviation and variance of the age of the project participants. Shows the mean, median, standard deviation and variance of the ages of the project participants.

N	Valid	89
	Loss	0
Mean		22,00
Median		21,00
Standard deviation		3,736
Variance		13,955

Table S15. Shows the frequency and percentage of the different ages of the project participants, as well as the standard deviation.

		Frequency	Percentage	Valid percentage	Cumulative percentage
Valid	20	30	33,7	33,7	33,7
	21	26	29,2	29,2	62,9
	22	20	22,5	22,5	85,4
	23	4	4,5	4,5	89,9
	25	1	1,1	1,1	91,0
	27	2	2,2	2,2	93,3
	29	3	3,4	3,4	96,6
	32	1	1,1	1,1	97,8
	36	1	1,1	1,1	98,9
	46	1	1,1	1,1	100,0
	Total	89	100,0	100,0	

Figure S2. A histogram showing the frequency of the different ages of the students.

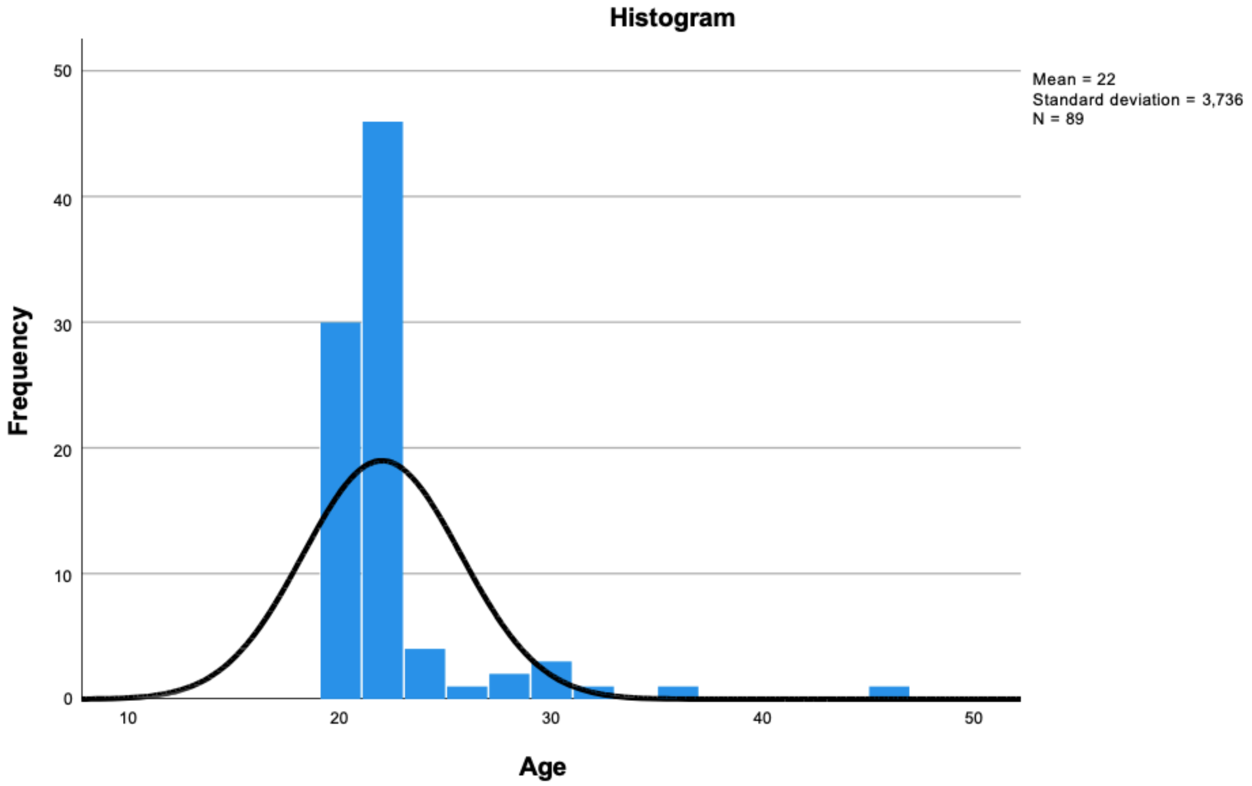

Table S16. Association between teacher presentation and gender. The table shows the relationship between the gender of the teacher and their presentation, distinguishing between failure and perfection. It includes observed and expected counts and adjusted residuals. There is a slight overrepresentation of females in the 'Perfect' category and of males in the 'Some failure' category, although the differences are minimal. The letters in the subscripts indicate subsets of gender categories whose column proportions do not differ significantly from each other at the 5% significance level.

			Gender		
			Female	Male	Total
Teacher presentation	Minor error	Count	0_{a}	1_{a}	1
		Expected count	,7	,3	1,0
		Corrected residue	-1,6	1,6	
	Correctly	Count	63_{a}	25_{a}	88
		Expected count	62,3	25,7	88,0
		Corrected residue	1,6	-1,6	
Total		Count	63	26	89
		Expected count	63,0	26,0	89,0

Each subscript letter denotes a subset of Sex categories whose column proportions do not differ significantly from each other at the .05 level

Table S17. Statistical analysis of the comparison of adequate teacher presentation by gender using chi-square tests. This table shows the results of the independence test applied to Table S16, using tests such as Pearson's chi-square, continuity correction, likelihood ratio and Fisher's exact test. The p-value (0.117) indicates that the null hypothesis is not rejected at the 0.05 level, meaning there is no significant association between the variables.

	Value	gl	Asymptotic significance (two-tailed)	Exact meaning (bilateral)	Exact meaning (unilateral)
Pearson's chi-square	2,451[a]	1	,117		
Continuity correction[b]	,211	1	,646		
Likelihood ratio	2,489	1	,115		
Fisher's exact test				,292	,292
Valid number of cases	89				

a. 2 boxes (50.0%) have expected a count less than 5. The minimum expected count is 0.29.
b. It has only been calculated for a 2x2 table.

Table S18.Symmetric Measures for the Association between Variables (Teacher presentation vs Gender). This table presents the Phi and Cramer's V coefficients, which are used to evaluate the strength and direction of the relationship between the variables analysed in the previous table. Both values (0.166) indicate a weak relationship, and the approximate significance level (p = 0.117) confirms that there is no statistically significant association at the 5% level. The analysis is based on 89 valid cases.

		Value	Approximate meaning
Nominal by Nominal	Phi	-,166	,117
	Cramer's V	,166	,117
Number of valid cases		89	

Table S19. Relationship between Workshop Presentation: Title and Gender. This cross-tabulation table shows the distribution of females and males in the 'No performance', 'Some failure' and 'Perfect' categories with regard to the presentation of the workshop title. Observed counts, expected counts and corrected residuals are included. The data indicate a slight overrepresentation of females in 'Some failure' and males in 'Perfect', although there are no significant differences.

Note on subscript: The letters indicate subsets of categories whose proportions do not differ significantly from each other at the 0.05 level.

			Gender		
			Female	Male	Total
Presentation of the workshop: Title.	Minor error	Count	4a	1a	5
		Expected count	3,5	1,5	5,0
		Corrected residue	,5	-,5	
	Omitted	Count	2a	1a	3
		Expected count	2,1	,9	3,0
		Corrected residue	-,2	,2	
	Correctly	Count	57a	24a	81
		Expected count	57,3	23,7	81,0
		Corrected residue	-,3	,3	
Total		Count	63	26	89
		Expected count	63,0	26,0	89,0

Table S20. Statistical analysis of the previous table using chi-square tests. This table presents the results of the independence test applied to Table S20. The chi-squared value (p = 0.888) indicates that the null hypothesis is not rejected; therefore, there is no significant association between gender and the presentation of the workshop title.

	Value	gl	Asymptotic significance (two-tailed)
Pearson's chi-square	,237[a]	2	,888
Likelihood ratio	,252	2	,882
Valid number of cases	89		

a. 4 boxes (66.7%) have expected a count lower than 5. The minimum expected count is 0.88.

Table S21. Symmetric Measures for the Association between Variables. Cramer's Phi and V coefficients (both equal 0.052) are shown to assess the strength of the association between the variables. The values, together with the significance (p = 0.888), confirm that the relationship is practically non-existent. The analysis is based on 89 valid cases.

		Value	Approximate meaning
Nominal by Nominal	Phi	,052	,888
	Cramer's V	,052	,888
Number of valid cases			

Table S22. Relationship between Definition of objectives and Gender. The cross-tabulation table shows the distribution of responses in the 'No performance', 'Some failure', 'Serious failure' and 'Perfect' categories, classified by gender. It includes observed counts, expected counts and corrected residuals. There is a slight overrepresentation of females in the 'Perfect' category and of males in the 'Some failure' category, but no significant differences.

			Gender		
			Female	Male	Total
Definition of objectives	Minor error	Count	3a	4a	7
		Expected count	5,0	2,0	7,0
		Corrected residue	-1,7	1,7	
	Major error	Count	2a	0a	2
		Expected count	1,4	,6	2,0
		Corrected residue	,9	-,9	
	Omitted	Count	3a	2a	5
		Expected count	3,5	1,5	5,0
		Corrected residue	-,5	,5	
	Correctly	Count	55a	20a	75
		Expected count	53,1	21,9	75,0
		Corrected residue	1,2	-1,2	
Total		Count	63	26	89
		Expected count	63,0	26,0	89,0

The letters indicate subsets of categories whose proportions do not differ significantly from each other at the 0.05 level.

Table S23. Statistical analysis of the previous table using chi-square tests. Presents the results of the independence test applied to the previous table. The chi-squared value ($p = 0.263$) indicates that the null hypothesis is not rejected; therefore, there is no significant association between gender and goal setting.

	Value	gl	Asymptotic significance (two-tailed)
Pearson's chi-square	3,982[a]	3	,263
Likelihood ratio	4,243	3	,236
Valid number of cases	89		

a. 6 boxes (75.0%) have expected a count lower than 5. The minimum expected count is 0.58.

Table S24. Symmetric measures for the association between variables. This includes Cramer's Phi and V coefficients (both equal 0.212), which are used to assess the strength of the association between the variables. The values, together with the significance level ($p = 0.263$), confirm that the relationship is weak and not significant. The analysis is based on 89 valid cases.

		Value	Approximate meaning
Nominal by Nominal	Phi	,212	,263
	Cramer's V	,212	,263
Number of valid cases			

Table S25. Relationship between performance of the technique in non-real time and gender. The cross-tabulation shows the distribution of responses by gender in the 'Does not perform', 'Some failure', and 'Perfect' categories with respect to execution of the technique in non-real time. It includes observed, expected and corrected residual counts. No significant differences between genders are observed.

Each subscript letter denotes a subset of Sex categories whose column proportions do not differ significantly from each other at the .05 level.

			Gender		
			Female	Male	Total Female
Carrying out the technique in non-real time	Minor error	Count	8_a	2_a	10
		Expected count	7,1	2,9	10,0
		Corrected residue	,7	-,7	
	Omitted	Count	0_a	1_a	1
		Expected count	,7	,3	1,0
		Corrected residue	-1,6	1,6	
	Correctly	Count	55_a	23_a	78
		Expected count	55,2	22,8	78,0
		Corrected residue	-,2	,2	
Total		Count	63	26	89
		Expected count	63,0	26,0	89,0

Table S26. Statistical analysis of the previous table using chi-square tests. Presents the results of the independence test applied to the previous table. The chi-squared value (p = 0.242) indicates that the null hypothesis is not rejected; therefore, there is no significant association between gender and performing the technique in non-real time.

	Value	gl	Asymptotic significance (two-tailed)
Pearson's chi-square	2,836[a]	2	,242
Likelihood ratio	2,906	2	,234
Valid number of cases	89		

a. 3 boxes (50.0%) have expected a count lower than 5. The minimum expected count is 0.29.

Table S27. Symmetric Measures for the Association between Variables. This includes Cramer's Phi and V coefficients (both equal 0.179), which are used to assess the strength of the association between the variables. The values, together with the significance (p = 0.242), confirm that the relationship is weak and not significant. The analysis is based on 89 valid cases.

		Value	Approximate meaning
Nominal by Nominal	Phi	,179	,242
	Cramer's V	,179	,242
Number of valid cases			

Table S28. Relationship between Allowing the Student to Ask All Questions and Gender. The cross-tabulation shows the distribution of responses by gender in the 'Does not do', 'Some failure' and 'Perfect' categories with regard to the possibility of the student asking all their questions. It includes observed and expected counts and corrected residuals. No significant gender differences are observed.

			Gender		
			Female	Male	Total
Allows the student to consult all the doubts	Minor error	Count	4_a	1_a	5
		Expected count	3,5	1,5	5,0
		Corrected residue	,5	-,5	
	Omitted	Count	1_a	0_a	1
		Expected count	,7	,3	1,0
		Corrected residue	,6	-,6	
	Correctly	Count	58_a	25_a	83
		Expected count	58,8	24,2	83,0
		Corrected residue	-,7	,7	
Total		Count	63	26	89
		Expected count	63,0	26,0	89,0

Each subscript letter denotes a subset of Sex categories whose column proportions do not differ significantly from each other at the .05 level

Table S29. Statistical analysis of the previous table using chi-square tests. Presents the results of the independence test applied to the previous table. The chi-squared value (p = 0.722) indicates that the null hypothesis is not rejected, meaning there is no significant association between gender and the likelihood of raising questions.

	Value	gl	Asymptotic significance (two-tailed)
Pearson's chi-square	,651[a]	2	,722
Likelihood ratio	,945	2	,623
Valid number of cases	89		

a. 4 boxes (66.7%) have expected a count lower than 5. The minimum expected count is 0.29.

Table S30. Symmetric Measures for the Association between Variables. This includes Cramer's Phi and V coefficients (both equal 0.086), which are used to assess the strength of the association between the variables. The values, together with the significance (p = 0.722), confirm that the relationship is very weak and not significant. The analysis is based on 89 valid cases.

		Value	Approximate meaning
Nominal by Nominal	Phi	,086	,722
	Cramer's V	,086	,722
Number of valid cases			

Table S31. Relationship between real-time technique performance and sex. The cross-tabulation shows the gender distribution in the "Does not perform", "Some errors" and "Perfect" categories with respect to the real-time execution of the technique. It includes observed and expected counts, as well as corrected residuals. There is a slight overrepresentation of men in the 'Some errors' category, but no significant differences were observed.

Each subscript letter denotes a subset of Sex categories whose column proportions do not differ significantly from each other at the .05 level.

			Gender		
			Female	Male	Total
Carrying out the technique in real time	Minor error	Count	14_a	9_a	23
		Expected count	16,3	6,7	23,0
		Corrected residue	-1,2	1,2	
	Omitted	Count	4_a	0_a	4
		Expected count	2,8	1,2	4,0
		Corrected residue	1,3	-1,3	
	Correctly	Count	45_a	17_a	62
		Expected count	43,9	18,1	62,0
		Corrected residue	,6	-,6	
Total		Count	63	26	89
		Expected count	63,0	26,0	89,0

Table S32. Statistical analysis of the previous table using chi-square tests. This presents the results of the independence test applied to the previous table. The chi-squared value (p = 0.242) indicates that the null hypothesis is not rejected, meaning there is no significant association between sex and performance of the real-time technique.

	Value	gl	Asymptotic significance (two-tailed)
Pearson's chi-square	2,841[a]	2	,242
Likelihood ratio	3,896	2	,143
Valid number of cases	89		

a. 2 boxes (33.3%) have expected a count lower than 5. The minimum expected count is 1.17.

Table S33. Symmetric Measures for the Association between Variables. This includes the Phi and Cramer's V coefficients (both equal 0.179), which are used to assess the strength of the association between the variables. The values, along with the significance (p = 0.242), confirm that the relationship is weak and not significant. The analysis is based on 89 valid cases.

		Value	Approximate meaning
Nominal by Nominal	Phi	,179	,242
	Cramer's V	,179	,242
Number of valid cases			

Table S34. Relationship between Summary with Definition of Key Points and Gender. The cross-tabulation shows the gender distribution in the 'Does not perform', 'Some failure', and 'Perfect' categories with respect to the definition of key points in the summary. It includes observed, expected and corrected residual counts. There is a slight overrepresentation of men in the 'Some failure' category and of women in the 'Perfect' category, with no significant differences.

Each subscript letter denotes a subset of Sex categories whose column proportions do not differ significantly from each other at the .05 level.

			Gender		
			Female	Male	Total
Summary with definition of key points	Minor error	Count	15_a	10_a	25
		Expected count	17,7	7,3	25,0
		Corrected residue	-1,4	1,4	
	Omitted	Count	7_a	2_a	9
		Expected count	6,4	2,6	9,0
		Corrected residue	,5	-,5	
	Correctly	Count	41_a	14_a	55
		Expected count	38,9	16,1	55,0
		Corrected residue	1,0	-1,0	
Total		Count	63	26	89
		Expected count	63,0	26,0	89,0

Each subscript letter denotes a subset of Sex categories whose column proportions do not differ significantly from each other at the .05 level

Table S35. Statistical analysis of the previous table using chi-square tests. This table presents the results of the independence test applied to the previous table. The chi-squared value (p = 0.369) indicates that the null hypothesis is not rejected, meaning that there is no significant association between sex and the definition of key points.

	Value	gl	Asymptotic significance (two-tailed)
Pearson's chi-square	1,995[a]	2	,369
Likelihood ratio	1,936	2	,380
Valid number of cases	89		

a. 1 boxes (16.7%) have expected a count lower than 5. The minimum expected count is 2.63.

Table S36. Symmetric Measures for the Association between Variable. This includes the Phi and Cramer's V coefficients (both equal 0.150), which are used to assess the strength of the association between the variables. The values, along with the significance (p = 0.369), confirm that the relationship is weak and not significant. The analysis is based on 89 valid cases.

		Value	Approximate meaning
Nominal by Nominal	Phi	,150	,369
	Cramer's V	,150	,369
Number of valid cases			

Table S37. Relationship between Performance of Feedback Techniques and Gender. The cross-tabulation shows the gender distribution in the 'Does Not Perform', 'Some Failure', and 'Perfect' categories regarding the application of feedback techniques. It includes observed and expected counts, as well as corrected residuals. No significant differences were observed between genders.

Each subscript letter denotes a subset of Sex categories whose column proportions do not differ significantly from each other at the .05 level.

			Gender		
			Female	Male	Total
Implementation of feedback techniques	Minor error	Count	6_a	3_a	9
		Expected count	6,4	2,6	9,0
		Corrected residue	-,3	,3	
	Omitted	Count	6_a	2_a	8
		Expected count	5,7	2,3	8,0
		Corrected residue	,3	-,3	
	Correctly	Count	51_a	21_a	72
		Expected count	51,0	21,0	72,0
		Corrected residue	,0	,0	
Total		Count	63	26	89
		Expected count	63,0	26,0	89,0

Table S38. Statistical analysis of the previous table using chi-square tests.

The results of the independence test applied to the previous table are presented. The chi-squared value (p = 0.931) indicates that the null hypothesis is not rejected, meaning there is no significant association between sex and the use of feedback techniques.

	Value	gl	Asymptotic significance (two-tailed)
Pearson's chi-square	,143[a]	2	,931
Likelihood ratio	,143	2	,931
Valid number of cases	89		

a. 1 Polling stations (33.3%) have expected a count of less than 5. The minimum expected count is 2.34.

Table S39. Symmetric measures for the association between variables. This table includes the Phi and Cramer's V coefficients (both equal 0.040), which are used to assess the strength of the association between the variables. The values, along with the significance (p = 0.931), confirm that the relationship is practically non-existent. The analysis is based on 89 valid cases.

		Value	Approximate meaning
Nominal by Nominal	Phi	,040	,931
	Cramer's V	,040	,931
Number of valid cases			

Table S40. Relationship between Invitation to Students to Perform the Technique and Gender. The cross-tabulation shows the gender distribution in the categories 'Does not perform', 'Some failure', and 'Perfect' with respect to the invitation to perform the technique. It includes observed and expected counts, as well as corrected residuals. Overrepresentation of males is observed in the 'Some failure' category, and overrepresentation of females is observed in the 'Perfect' category, with significant differences.

Each subscript letter denotes a subset of Sex categories whose column proportions do not differ significantly from each other at the .05 level.

			Gender		Total
			Female	Male	
Invitation to students to perform the technique	Minor error	Count	3a	5b	8
		Expected count	5,7	2,3	8,0
		Corrected residue	-2,2	2,2	
	Omitted	Count	5a	0a	5
		Expected count	3,5	1,5	5,0
		Corrected residue	1,5	-1,5	
	Correctly	Count	55a	21a	76
		Expected count	53,8	22,2	76,0
		Corrected residue	,8	-,8	
Total		Count	63	26	89
		Expected count	63,0	26,0	89,0

Table S41. Statistical analysis of the previous table using chi-square tests. The results of the independence test applied to the previous table are presented below. The chi-squared value (p = 0.040) indicates that the null hypothesis is rejected and that there is a significant association between sex and being invited to undergo the technique.

	Value	gl	Asymptotic significance (two-tailed)
Pearson's chi-square	6,442[a]	2	,040
Likelihood ratio	7,341	2	,025
Valid number of cases	89		

a. 1 polling stations (50.0%) have expected a count of less than 5. The minimum expected count is 1.46.

Table S42. Symmetric Measures for the Association between Variables. This table includes the Phi and Cramer's V coefficients (both equal 0.269), which are used to assess the strength of the association between the variables. The values, along with the significance (p = 0.040), confirm a weak but significant relationship. The analysis is based on 89 valid cases.

		Value	Approximate meaning
Nominal by Nominal	Phi	,269	,040
	Cramer's V	,269	,040
Number of valid cases			

Table S43. Relationship between Self-Criticism and Sex. The cross-tabulation shows the gender distribution in the 'Does Not Perform', 'Some Failure', and 'Perfect' categories with respect to self-criticism. It includes observed and expected counts, as well as corrected residuals. No significant differences are observed between genders.

Each subscript letter denotes a subset of Gender categories whose column proportions do not differ significantly from each other at the .05 level.

			Gender		
			Female	Male	Total
Self-critics	Minor error	Count	4_a	2_a	6
		Expected count	4,2	1,8	6,0
		Corrected residue	-,2	,2	
	Omitted	Count	7_a	1_a	8
		Expected count	5,7	2,3	8,0
		Corrected residue	1,1	-1,1	
	Correctly	Count	52_a	23_a	75
		Expected count	53,1	21,9	75,0
		Corrected residue	-,7	,7	
Total		Count	63	26	89
		Expected count	63,0	26,0	89,0

Table S44. Statistical analysis of the previous table using chi-square tests. This presents the results of the independence test applied to the previous table. The chi-squared value (p = 0.547) indicates that the null hypothesis is not rejected, meaning there is no significant association between sex and self-criticism.

	Value	gl	Asymptotic significance (two-tailed)
Pearson's chi-square	1,206[a]	2	,547
Likelihood ratio	1,394	2	,498
Valid number of cases	89		

a. 3 boxes (50.0%) have expected a count lower than 5. The minimum expected count is 1.75.

Table S45. Symmetric measures for the association between variables. This table includes the Phi and Cramer's V coefficients (both equal 0.116), which are used to assess the strength of the association between the variables. The values, along with the significance level (p = 0.547), confirm that the relationship is weak and not statistically significant. The analysis is based on 89 valid cases.

		Value	Approximate meaning
Nominal by Nominal	Phi	,116	,547
	Cramer's V	,116	,547
Number of valid cases			

Table S46. Relationship between course and participation in emergency care or PCR. Grade 3rd, 4th and 5th based on participation in emergency care or PCR (Yes/No). It includes observed and expected counts, percentages, and corrected residuals. There is an overrepresentation of the third grade in the 'No' category and the fourth grade in the 'Yes' category, with significant differences.

Note on subscript: Letters indicate subsets of categories whose proportions do not differ significantly from each other at the 0.05 level.

			Previously attended an emergency or CPR.		
			No	Yes	Total
Course	3	Count	38a	2b	40
		Expected count	33,3	6,7	40,0
		Percentage of total	42,7%	2,2%	44,9%
		Corrected residue	2,7	-2,7	
	4	Count	33a	12b	45
		Expected count	37,4	7,6	45,0
		Percentage of total	37,1%	13,5%	50,6%
		Corrected residue	-2,5	2,5	
	5	Count	3a	1a	4
		Expected count	3,3	,7	4,0
		Percentage of total	3,4%	1,1%	4,5%
		Corrected residue	-,4	,4	
Total		Count	74	15	89
		Expected count	74,0	15,0	89,0
		Percentage of total	83,1%	16,9%	100,0%

Table S47. Statistical analysis of the previous table using chi-square tests. The results of the independence test applied to the previous table are presented. The chi-square value (p = 0.026) indicates that the null hypothesis is rejected, suggesting a significant association between the course and participation in emergency care or PCR.

Note on subscript: Indicates that some cells have expected counts of less than 5, which may affect the robustness of the contrast.

	Value	gl	Asymptotic significance (two-tailed)
Pearson's chi-square	7,292[a]	2	,026
Likelihood ratio	8,162	2	,017
Valid number of cases	89		

Table S48. Symmetric Measures for the Association between Variables. This table includes the Phi and Cramer's V coefficients (both equal 0.286), which are used to assess the strength of the association between the variables. The values, along with the significance (p = 0.026), confirm a weak but significant relationship. The analysis is based on 89 valid cases.

		Value	Approximate meaning
Nominal by Nominal	Phi	,286	,026
	Cramer's V	,286	,026
Number of valid cases		89	

Table S49. Relationship between course and adequate teacher presentation. This crosstab shows the distribution by grade (3rd, 4th and 5th) of the 'Some Failure' and 'Perfect' categories with regard to adequate teacher presentation. It includes observed and expected counts, percentages, and corrected residuals. No significant differences are observed between grades.

Note on subscript: Letters indicate subsets of categories whose proportions do not differ significantly from each other at the 0.05 level.

			Teacher presentation		
			Minor error	Correctly	Total
Course	3	Count	1_a	39_a	40
		Expected count	,4	39,6	40,0
		Percentage of total	1,1%	43,8%	44,9%
		Corrected residue	1,1	-1,1	
	4	Count	0_a	45_a	45
		Expected count	,5	44,5	45,0
		Percentage of total	0,0%	50,6%	50,6%
		Corrected residue	-1,0	1,0	
	5	Count	0_a	4_a	4
		Expected count	,0	4,0	4,0
		Percentage of total	0,0%	4,5%	4,5%
		Corrected residue	-,2	,2	
Total		Count	1	88	89
		Expected count	1,0	88,0	89,0
		Percentage of total	1,1%	98,9%	100,0%

Table S50. Statistical analysis of the previous table using chi-square tests. This presents the results of the independence test applied to the previous table. The chi-squared value (p = 0.538) indicates that the null hypothesis is not rejected, meaning there is no significant association between the course and the teacher's adequate presentation.

Note on subscript:Indicates that some cells have expected counts of less than 5, which may affect the robustness of the contrast.

	Value	gl	Asymptotic significance (two-tailed)
Pearson’s chi-square	1,239[a]	2	,538
Likelihood ratio	1,613	2	,446
Valid number of cases	89		

Table S51. Symmetric measures for the association between variables. This table includes the Phi and Cramer's V coefficients (both equal 0.118), which are used to assess the strength of the association between the variables. The values, along with the significance level (p = 0.538), confirm that the relationship is weak and not statistically significant. The analysis is based on 89 valid cases.

		Value	Approximate meaning
Nominal por Nominal	Phi	,118	,538
	Cramer's V	,118	,538
Valid number of cases		89	

Table S52. Relationship between Course and Appropriate Workshop Presentation: Title. The crosstab shows the distribution by grade (3rd, 4th, and 5th) in the categories Did Not Perform, Some Failure, and Perfect with respect to the presentation of the workshop title. It includes observed and expected counts, percentages, and corrected residuals. No significant differences are observed between grades.

Note on subscript: Letters indicate subsets of categories whose proportions do not differ significantly from each other at the 0.05 level.

			Presentation of the workshop: Title.			
			Minor error	Omitted	Correctly	Total
Course	3	Count	1a	0a	39a	40
		Expected count	2,2	1,3	36,4	40,0
		Percentage of total	1,1%	0,0%	43,8%	44,9%
		Corrected residue	-1,2	-1,6	1,9	
	4	Count	3a	3a	39a	45
		Expected count	2,5	1,5	41,0	45,0
		Percentage of total	3,4%	3,4%	43,8%	50,6%
		Corrected residue	,4	1,7	-1,4	
	5	Count	1a	0a	3a	4
		Expected count	,2	,1	3,6	4,0
		Percentage of total	1,1%	0,0%	3,4%	4,5%
		Corrected residue	1,7	-,4	-1,1	
Total		Count	5	3	81	89
		Expected count	5,0	3,0	81,0	89,0
		Percentage of total	5,6%	3,4%	91,0%	100,0%

Table S53. Statistical analysis of the previous table using chi-square tests. This presents the results of the independence test applied to the previous table. The chi-squared value ($p \approx 0.148$) indicates that the null hypothesis is not rejected, meaning there is no significant association between the course and the presentation of the workshop title.

Note on subscript: Indicates that some cells have expected counts of less than 5, which may affect the robustness of the contrast.

	Value	gl	Asymptotic significance (two-tailed)
Pearson's chi-square	6,779[a]	4	,148
Likelihood ratio	6,881	4	,142
Valid number of cases	89		

Table S54. Symmetric Measures for the Association between Variables. This table includes the Phi and Cramer's V coefficients (which have low values) in order to assess the strength of the association between the variables. The results, along with the significance (p ≈ 0.148), confirm that the relationship is weak and not significant. The analysis is based on 89 valid cases.

		Value	Approximate meaning
Nominal por Nominal	Phi	,276	,148
	Cramer's V	,195	,148
Valid number of cases		89	

Table S55. Relationship between Course and Objective Definition. The crosstab shows the distribution by grade (3rd, 4th, and 5th) in the categories Does Not Perform, Some Failure, Serious Failure, and Perfect with respect to the definition of objectives. It includes observed and expected counts, percentages, and corrected residuals. No significant differences are observed between grades.

Note on subscript: Letters indicate subsets of categories whose proportions do not differ significantly from each other at the 0.05 level.

			Definition of objectives				
			Minor error	Fallo grave	Omitted	Correctly	Total
Course	3	Count	2_{a}	1_{a}	2_{a}	35_{a}	40
		Expected count	3,1	,9	2,2	33,7	40,0
		Percentage of total	2,2%	1,1%	2,2%	39,3%	44,9%
		Corrected residue	-,9	,1	-,2	,8	
	4	Count	4_{a}	0_{a}	3_{a}	38_{a}	45
		Expected count	3,5	1,0	2,5	37,9	45,0
		Percentage of total	4,5%	0,0%	3,4%	42,7%	50,6%
		Corrected residue	,4	-1,4	,4	,0	
	5	Count	$1_{a, b}$	1_{b}	$0_{a, b}$	2_{a}	4
		Expected count	,3	,1	,2	3,4	4,0
		Percentage of total	1,1%	1,1%	0,0%	2,2%	4,5%
		Corrected residue	1,3	3,1	-,5	-1,9	
Total		Count	7	2	5	75	89
		Expected count	7,0	2,0	5,0	75,0	89,0
		Percentage of total	7,9%	2,2%	5,6%	84,3%	100,0%

Table S56. Statistical analysis of the previous table using chi-square tests. This presents the results of the independence test applied to the previous table. The chi-square value indicates that the null hypothesis is not rejected, meaning there is no significant association between the course and goal setting.

Note on subscript: Indicates that some cells have expected counts of less than 5, which may affect the robustness of the contrast.

	Value	gl	Asymptotic significance (two-tailed)
Pearson's chi-square	13,155[a]	6	,041
Likelihood ratio	7,775	6	,255
Valid number of cases	89		

Table S57. Symmetric Measures for the Association between Variables. This table includes the Phi and Cramer's V coefficients, which are used to assess the strength of the association between variables. The values, along with the significance level, confirm that the relationship is weak and not significant. The analysis is based on 89 valid cases.

		Value	Approximate meaning
Nominal por Nominal	Phi	,384	,041
	Cramer's V	,272	,041
Valid number of cases		89	

Table S58. Relationship between Course and Non-Real-Time Technique Execution. The crosstab shows the distribution by grade (3rd, 4th, and 5th) in the categories Did Not Perform, Some Error, and Perfect with respect to execution of the non-real-time technique. It includes observed and expected counts, percentages, and corrected residuals. No significant differences were observed between grades.

Note on subscript: Letters indicate subsets of categories whose proportions do not differ significantly from each other at the 0.05 level.

			Carrying out the technique in non-real time			
			in	Omitted	Correctly	Total
Course	3	Count	4_a	0_a	36_a	40
		Expected count	4,5	,4	35,1	40,0
		Percentage of total	4,5%	0,0%	40,4%	44,9%
		Corrected residue	-,3	-,9	,6	
	4	Count	6_a	1_a	38_a	45
		Expected count	5,1	,5	39,4	45,0
		Percentage of total	6,7%	1,1%	42,7%	50,6%
		Corrected residue	,6	1,0	-,9	
	5	Count	0_a	0_a	4_a	4
		Expected count	,4	,0	3,5	4,0
		Percentage of total	0,0%	0,0%	4,5%	4,5%
		Corrected residue	-,7	-,2	,8	
Total		Count	10	1	78	89
		Expected count	10,0	1,0	78,0	89,0
		Percentage of total	11,2%	1,1%	87,6%	100,0%

Table S59. Statistical analysis of the previous table using chi-square tests. This presents the results of the independence test applied to the previous table. The chi-squared value (p = 0.772) indicates that the null hypothesis is not rejected; in other words, there is no significant association between the course and the performance of the non-real-time technique.

Note on subscript: Indicates that 66.7% of the boxes have expected counts of less than 5 (minimum expected = 0.4).

	Value	gl	Asymptotic significance (two-tailed)
Pearson's chi-square	1,805[a]	4	,772
Likelihood ratio	2,630	4	,621
Valid number of cases	89		

Table S60. Symmetric Measures for the Association between Variables. This table includes the Phi (0.142) and Cramer's V (0.101) coefficients, which are used to assess the strength of the association between the variables. The values, along with the significance (p = 0.772), confirm that the relationship is weak and not significant. The analysis is based on 89 valid cases.

		Value	Approximate meaning
Nominal por Nominal	Phi	,142	,772
	Cramer's V	,101	,772
Valid number of cases		89	

Table S61. Relationship between Course and Allowing Students to Consult All Questions. The crosstab shows the distribution by grade (3rd, 4th, and 5th) in the categories of 'Does not complete', 'Some errors', and 'Perfect' with respect to the ability to answer all questions. It includes observed and expected counts, percentages, and corrected residuals. There is an overrepresentation of the Year 3 students in the 'Some errors' category and Year 4 students in the 'Perfect' category, although the evidence is not conclusive.

Note on subscript: Letters indicate subsets of categories whose proportions do not differ significantly from each other at the 0.05 level.

			Allows the student to consult all the doubts			
			Minor error	Omitted	Correctly	Total
Course	3	Count	5_a	0_b	35_b	40
		Expected count	2,2	,4	37,3	40,0
		Percentage of total	5,6%	0,0%	39,3%	44,9%
		Corrected residue	2,5	-,9	-2,0	
	4	Count	0_a	1_b	$44_{a, b}$	45
		Expected count	2,5	,5	42,0	45,0
		Percentage of total	0,0%	1,1%	49,4%	50,6%
		Corrected residue	-2,3	1,0	1,7	
	5	Count	0_a	0_a	4_a	4
		Expected count	,2	,0	3,7	4,0
		Percentage of total	0,0%	0,0%	4,5%	4,5%
		Corrected residue	-,5	-,2	,6	
Total		Count	5	1	83	89
		Expected count	5,0	1,0	83,0	89,0
		Percentage of total	5,6%	1,1%	93,3%	100,0%

Table S62. Statistical analysis of the previous table using chi-square tests. This presents the results of the independence test applied to the previous table. The chi-squared value (p = 0.118) suggests that the null hypothesis cannot be rejected. However, the likelihood ratio (p = 0.047) indicates a possible weak association between the course and the likelihood of asking questions.

Note on subscript: Indicates that 77% of the boxes have expected counts of less than 5 (minimum expected = 0.4).

	Value	gl	Asymptotic significance (two-tailed)
Pearson's chi-square	7,363[a]	4	,118
Likelihood ratio	9,623	4	,047
Valid number of cases	89		

Table S63. Symmetric measures for the association between variables. This table includes the Phi (0.288) and Cramer's V (0.203) coefficients, which are used to evaluate the strength of the relationship between the variables. The values, along with the significance level (p = 0.118), confirm that the relationship is weak and not significant according to the main criterion. The analysis is based on 89 valid cases.

		Value	Approximate meaning
Nominal por Nominal	Phi	,288	,118
	Cramer's V	,203	,118
Valid number of cases		89	

Table S64. Relationship between Course and Real-Time Technique Execution. The crosstab shows the distribution by grade (3rd, 4th, and 5th) in the Does not perform, Some errors, and Perfect categories with respect to real-time technique execution. It includes observed and expected counts, percentages, and corrected residuals. There is an overrepresentation of the 3rd grade in 'Perfect' and the 4th grade in 'Some errors', with significant differences.

Note on subscript: Letters indicate subsets of categories whose proportions do not differ significantly from each other at the 0.05 level.

			Carrying out the technique in real time			
			Minor error	Omitted	Correctly	Total
Course	3	Count	7a	0a	33a	40
		Expected count	10,3	1,8	27,9	40,0
		Percentage of total	7,9%	0,0%	37,1%	44,9%
		Corrected residue	-1,6	-1,8	2,4	
	4	Count	14a	4a	27a	45
		Expected count	11,6	2,0	31,3	45,0
		Percentage of total	15,7%	4,5%	30,3%	50,6%
		Corrected residue	1,1	2,0	-2,0	
	5	Count	2a	0a	2a	4
		Expected count	1,0	,2	2,8	4,0
		Percentage of total	2,2%	0,0%	2,2%	4,5%
		Corrected residue	1,1	-,4	-,9	
Total		Count	23	4	62	89
		Expected count	23,0	4,0	62,0	89,0
		Percentage of total	25,8%	4,5%	69,7%	100,0%

Table S65. Statistical analysis of the previous table using chi-square tests. The results of the independence test applied to the previous table are presented. The chi-squared value (p = 0.086) suggests that the null hypothesis cannot be rejected. However, the likelihood ratio (p = 0.048) indicates a possible weak association between the course and the use of the real-time technique.

Note on subscript: Indicates that 55% of the boxes have expected counts of less than 5 (expected minimum = 0.18).

	Value	gl	Asymptotic significance (two-tailed)
Pearson's chi-square	8,146[a]	4	,086
Likelihood ratio	9,606	4	,048
Valid number of cases	89		

Table S66. Symmetric Measures for the Association between Variables. This table includes the Phi (0.303) and Cramer's V (0.214) coefficients, which are used to assess the strength of the association between the variables. The values, along with the significance (p = 0.086), confirm that the relationship is weak and not significant according to the main criterion. The analysis is based on 89 valid cases.

		Value	Approximate meaning
Nominal por Nominal	Phi	,303	,086
	Cramer's V	,214	,086
Valid number of cases		89	

Table S67. Relationship between Course and Summary with Definition of Key Points. This crosstab shows the distribution by grade (3rd, 4th and 5th) of the categories Does Not Perform, Some Failure and Perfect, with respect to the definition of key points in the summary. It includes observed and expected counts, percentages, and corrected residuals. There is an overrepresentation of 3rd graders in the 'Some Failure' category and an underrepresentation in the 'Does Not Perform' category, with significant differences.

Note on subscript: Letters indicate subsets of categories whose proportions do not differ significantly from each other at the 0.05 level.

			Summary with definition of key points			
			Minor error	Omitted	Correctly	Total
Course	3	Count	15a	0b	25a	40
		Expected count	11,2	4,0	24,7	40,0
		Percentage of total	16,9%	0,0%	28,1%	44,9%
		Corrected residue	1,8	-2,9	,1	
	4	Count	7a	9b	29a	45
		Expected count	12,6	4,6	27,8	45,0
		Percentage of total	7,9%	10,1%	32,6%	50,6%
		Corrected residue	-2,7	3,1	,5	
	5	Count	3a	0a	1a	4
		Expected count	1,1	,4	2,5	4,0
		Percentage of total	3,4%	0,0%	1,1%	4,5%
		Corrected residue	2,1	-,7	-1,5	
Total		Count	25	9	55	89
		Expected count	25,0	9,0	55,0	89,0
		Percentage of total	28,1%	10,1%	61,8%	100,0%

Table S68. Statistical analysis of the previous table using chi-square tests. This presents the results of the independence test applied to the previous table. The chi-square value (p = 0.002) indicates that the null hypothesis is rejected and that there is a significant association between the course and the definition of key points.

Note on subscript: Indicates that several cells have expected counts of less than 5, which may affect the robustness of the contrast.

	Value	gl	Asymptotic significance (two-tailed)
Pearson's chi-square	16,642[a]	4	,002
Likelihood ratio	19,749	4	<,001
Valid number of cases	89		

Table S69. Symmetric Measures for the Association between Variables. This table includes the Phi (0.432) and Cramer's V (0.306) coefficients, which are used to assess the strength of the association between the variables. The values, along with the significance (p = 0.002), confirm a moderate and significant relationship. The analysis is based on 89 valid cases.

		Value	Approximate meaning
Nominal por Nominal	Phi	,432	,002
	Cramer's V	,306	,002
Valid number of cases		89	

Table S70. Relationship between Course and Performance of Feedback Techniques. The crosstab shows the distribution by grade (3rd, 4th, and 5th) in the Does Not Perform, Some Failure, and Perfect categories regarding the application of feedback techniques. It includes observed and expected counts, percentages, and corrected residuals. No significant differences are observed between grades.

Note on subscript: Letters indicate subsets of categories whose proportions do not differ significantly from each other at the 0.05 level.

			Implementation of feedback techniques			
			Minor error	Omitted	Correctly	Total
Course	3	Count	4_a	2_a	34_a	40
		Expected count	4,0	3,6	32,4	40,0
		Percentage of total	4,5%	2,2%	38,2%	44,9%
		Corrected residue	,0	-1,2	,9	
	4	Count	4_a	5_a	36_a	45
		Expected count	4,6	4,0	36,4	45,0
		Percentage of total	4,5%	5,6%	40,4%	50,6%
		Corrected residue	-,4	,7	-,2	
	5	Count	1_a	1_a	2_a	4
		Expected count	,4	,4	3,2	4,0
		Percentage of total	1,1%	1,1%	2,2%	4,5%
		Corrected residue	1,0	1,1	-1,6	
Total		Count	9	8	72	89
		Expected count	9,0	8,0	72,0	89,0
		Percentage of total	10,1%	9,0%	80,9%	100,0%

Table S71. Statistical analysis of the previous table using chi-square tests. This presents the results of the independence test applied to previous table. The chi-squared value indicates that the null hypothesis is not rejected, meaning that there is no significant association between the course and the use of feedback techniques.

Note on subscript: Indicates that some cells have expected counts of less than 5, which may affect the robustness of the contrast.

	Value	gl	Asymptotic significance (two-tailed)
Pearson's chi-square	3,578[a]	4	,466
Likelihood ratio	3,142	4	,534
Valid number of cases	89		

Table S72. Symmetric Measures for the Association between Variables. This table includes the Phi and Cramer's V coefficients, which are used to assess the strength of the association between variables. The values, along with the significance level, confirm that the relationship is weak and not significant. The analysis is based on 89 valid cases.

		Value	Approximate meaning
Nominal por Nominal	Phi	,201	,466
	Cramer's V	,142	,466
Valid number of cases		89	

Table S73. Relationship between Course and Invitation to Students to Perform the Technique. The crosstab shows the distribution by grade (3rd, 4th, and 5th) in the categories Did not perform, Some errors, and Perfect with respect to the invitation to perform the technique. It includes observed and expected counts, percentages, and corrected residuals. There is an overrepresentation of the third grade in 'Some errors' and the fourth grade in 'Perfect', with significant differences.

Note on subscript: Letters indicate subsets of categories whose proportions do not differ significantly from each other at the 0.05 level.

			Invitation to students to perform the technique			Total
			Minor error	Omitted	Correctly	
Course	3	Count	7_{a}	$2_{a, b}$	31_{b}	40
		Expected count	3,6	2,2	34,2	40,0
		Percentage of total	7,9%	2,2%	34,8%	44,9%
		Corrected residue	2,5	-,2	-1,9	
	4	Count	0_{a}	3_{b}	42_{b}	45
		Expected count	4,0	2,5	38,4	45,0
		Percentage of total	0,0%	3,4%	47,2%	50,6%
		Corrected residue	-3,0	,4	2,1	
	5	Count	1_{a}	0_{a}	3_{a}	4
		Expected count	,4	,2	3,4	4,0
		Percentage of total	1,1%	0,0%	3,4%	4,5%
		Corrected residue	1,1	-,5	-,6	
Total		Count	8	5	76	89
		Expected count	8,0	5,0	76,0	89,0
		Percentage of total	9,0%	5,6%	85,4%	100,0%

Table S74. Statistical analysis of the previous table using chi-square tests. The results of the independence test applied to the previous table are presented. The chi-squared value (p = 0.051) indicates a near-significant association, whereas the likelihood ratio (p = 0.013) suggests a significant relationship between the course and the invitation to perform the technique.

Note on subscript:Indicates that several cells have expected counts of less than 5, which may affect the robustness of the contrast.

	Value	gl	Asymptotic significance (two-tailed)
Pearson's chi-square	9,424[a]	4	,051
Likelihood ratio	12,610	4	,013
Valid number of cases	89		

Table S75. Symmetric Measures for the Association between Variables. This table includes the Phi (0.325) and Cramer's V (0.230) coefficients, which are used to assess the strength of the association between the variables. The values, together with the significance level of p = 0.051, confirm a weak relationship that is close to being statistically significant. The analysis is based on 89 valid cases.

		Value	Approximate meaning
Nominal por Nominal	Phi	,325	,051
	Cramer's V	,230	,051
Valid number of cases		89	

Table S76. Relationship between Course and Self-Criticism. The cross-tabulation shows the distribution by grade (3rd, 4th, and 5th) in the Does Not Perform, Some Errors, and Perfect categories with respect to self-criticism. It includes observed and expected counts, percentages, and corrected residuals. There is a slight overrepresentation of the third grade in the 'Some Errors' and 'Does Not Perform' categories, with no significant differences.

Note on subscript: Letters indicate subsets of categories whose proportions do not differ significantly from each other at the 0.05 level.

			Self-critics			
			Minor error	Omitted	Correctly	Total
Course	3	Count	4_a	5_a	31_a	40
		Expected count	2,7	3,6	33,7	40,0
		Percentage of total	4,5%	5,6%	34,8%	44,9%
		Corrected residue	1,1	1,0	-1,6	
	4	Count	2_a	2_a	41_a	45
		Expected count	3,0	4,0	37,9	45,0
		Percentage of total	2,2%	2,2%	46,1%	50,6%
		Corrected residue	-,9	-1,5	1,8	
	5	Count	0_a	1_a	3_a	4
		Expected count	,3	,4	3,4	4,0
		Percentage of total	0,0%	1,1%	3,4%	4,5%
		Corrected residue	-,6	1,1	-,5	
Total		Count	6	8	75	89
		Expected count	6,0	8,0	75,0	89,0
		Percentage of total	6,7%	9,0%	84,3%	100,0%

Table S77. Statistical analysis of the previous table using chi-square tests.This presents the results of the independence test applied to the previous table. The chi-square value indicates that the null hypothesis is not rejected, meaning there is no significant association between grade level and self-criticism.

Note on subscript: Indicates that some cells have expected counts of less than 5, which may affect the robustness of the contrast.

	Value	gl	Asymptotic significance (two-tailed)
Pearson's chi-square	4,483[a]	4	,345
Likelihood ratio	4,523	4	,340
Valid number of cases	89		

Table S78. Symmetric Measures for the Association between Variables. This table includes the Phi and Cramer's V coefficients, which are used to assess the strength of the association between variables. The values, along with the significance level, confirm that the relationship is weak and not significant. The analysis is based on 89 valid cases.

		Value	Approximate meaning
Nominal por Nominal	Phi	,224	,345
	Cramer's V	,159	,345
Valid number of cases		89	
